# Exclusive Breastfeeding and Normative Belief among Rural Mothers in Ethiopia, 2019: A Cross-Sectional Survey Embedded with Qualitative Design

**DOI:** 10.1155/2021/5587790

**Published:** 2021-03-16

**Authors:** Wolde Melese Ayele

**Affiliations:** Department of Epidemiology and Biostatistics, College of Medicine and Health Sciences, Wollo University, Dessie, Ethiopia

## Abstract

**Background:**

Exclusive breastfeeding has an irrepressible benefit to a child. However, the practice is still low with salient factors in Ethiopia. Therefore, this study aimed to assess exclusive breastfeeding practice and normative beliefs among mothers who have children less than two years of age in Ethiopia, 2019.

**Methods:**

A community-based cross-sectional study was conducted with a sample size of 423 in Ethiopia from March 12 to December 18, 2019. An interviewer-administered questionnaire was used to collect the data. Gender-matched six Focus Group Discussions were conducted. Semistructured guiding questions were used to carry out the discussion. The binary logistic regression model was used to determine the association between dependent and independent variables of the quantitative part.

**Results:**

The prevalence of exclusive breastfeeding practice was 77.5% (95% CI: 73.5, 81.5%). Married mothers (AOR = 2.57; 95% CI: 1.68, 5.65), mothers with antenatal care follow-up (AOR = 4.11; 95% CI: 2.66, 11.17), mothers who delivered at a health institution (AOR = 4.07; 95% CI: 2.99, 10.72), and mothers counseled during antenatal care (AOR = 1.96; 95% CI: 1.12, 4.73) had a positive association, whereas mothers who were unable to read and write (AOR = 0.11; 95% CI: 0.06, 0.99) and employed mothers (AOR = 0.22; 95% CI: 0.16, 0.56) were the variables that had a negative association with exclusive breastfeeding practice.

**Conclusions:**

Although the prevalence of exclusive breastfeeding was good when compared with other studies, rigorous interventions are needed to achieve the WHO recommendation of all infants should exclusively be breastfed. Marital status, educational status, occupation, antenatal care service, place of birth, and counseling of mothers during ANC were factors associated with the exclusive breastfeeding practice.

## 1. Introduction

Breastfeeding is the practice of a woman feeding an infant and a young child [1]. Breast milk has extreme products that a newborn needs [2].

Breast milk contains all the essential nutrients that a healthy child needs [3]. The World Health Organization (WHO) infant-feeding guideline recommended that all infants should be breastfed within one hour of birth and exclusively breastfed until six months of life. Optimal feeding of infants and children means exclusive breastfeeding from birth to six months. After six months of age, complementary foods are introduced in addition to breastfeeding [4].

Currently, only 39% of all infants are exclusively breastfed worldwide. The prevalence is about 30% in most developing world countries. Globally, because of inadequate breastfeeding practice in combination with high levels of diseases, more than 10 million children under five years of age die each year. Of this figure, 41% occur in sub-Saharan Africa and 34% in the South Asia region [5].

A cohort study carried out in Ghana revealed that 22% of neonatal deaths could be prevented if all infants were breastfed within the first hour of birth [6]. It has also been reported that exclusive breastfeeding from birth and until 6 months of age has the potential to prevent 13% of all deaths among children, aged less than 5 years, annually in developing countries [7]. A study in Addis Ababa showed that 60.0% of mothers gave prelacteal fluids and 32.0% infants less than four months were exclusively breastfed [8]. There is a wide range of variation in the practice of exclusive breastfeeding in developing countries, with the rates documented as Brazil 58%, Bangalore 40%, Iran 69%, Beruwala 15.5%, Lebanon 10.1%, Nigeria 20%, Bangladesh 34.5%, and Jordan 77%. In Ethiopia, 49% of infants were breastfed for the first six months, and 56.9% were exclusively breastfed for the first four months [9].

According to the 2016 EDHS, 58% in Ethiopia [10], 64.4% in Jimma [11], 67.2% in Bishoftu town [12], 18.0% in Dabat [13], and 41.8% in Tigray [14] were started on breastfeeding within the first one hour after birth. Similarly, studies in Debre Tabor [15], Enderta [16], Motta [17], Guba [18], and Gondar town [19] showed that 70.8%, 70.2%, 50.1%, 71.3%, and 35.9% of the children, respectively, were breastfed exclusively.

The salient factors associated with exclusive breastfeeding are maternal age, maternal level of education, maternal employment status, maternal knowledge, place of delivery, residence, culture, and household wealth status [11, 12, 15, 17, 19]. Despite these facts, there is no study conducted about EBF in the study area.

Therefore, this study aimed to assess the prevalence of exclusive breastfeeding and normative belief of mothers in Ethiopia. The findings of this study will be crucial for health service providers, nongovernmental organizations working on this field, policy makers, and program managers to design intervention strategies that may promote optimal breastfeeding practices in the country. Since the study is embedded with a qualitative design, it will explore hidden factors for future researchers and for an immediate intervention process.

## 2. Materials and Methods

### 2.1. Study Area and Participants

A community-based cross-sectional study was conducted from 12 March to 18 December 2019 in six districts of Northeastern Ethiopia. The study included mothers whose child was less than two years of age. The districts were selected using a simple random sampling lottery method among 21 districts.

The sample size was calculated using a single population proportion formula by considering the following assumptions: proportion (50.1%) of excessive breastfeeding from the study in Motta town [17], the 95% confidence level, margin of error (*d* = 5%), and 10% nonresponse rate. A simple random sampling technique was used to select 423 mothers to participate in the study. The sample size in each district was allocated proportionally for the expected mothers whose child was less than 2 year of age. This is from the central statistical agency population projection of Ethiopia by the district level. The actual age of the infant was determined by asking the mothers and/or reviewing the birth certificate. If the birth certificate was not available, only the mothers' recall was used to assure the infant's age. Only mothers of age 18 and above were included. Mothers who were too sick to feed their child exclusively and children who were unable to suck because of medical or other problems were excluded from the study.

Similarly, six (three among male and three among female parents) Focus Group Discussions (FGD) were conducted. The FGD was gender-matched among the eligible mothers in the quantitative study.

### 2.2. Data Collection Procedure

An interviewer-administered structured questionnaire was applied for data collection. The questionnaire was designed originally in English and translated to the local (Amharic) language. The questionnaire is adopted from Ethiopian Demographic and Health Survey (EDHS) 2016 [10] and from the previous similar studies [17, 20, 21]. As shown in supplementary 1, the questionnaire has four parts: sociodemographic characteristics, maternal and child health variables, breastfeeding related, and factors associated with exclusive breastfeeding. The questionnaire has not been previously published elsewhere. It was designed for this study. The questionnaire quality was checked by pretesting 5% of the samples. The pretest data were not included in the final model. Although it might introduce recall bias, the mothers' recall method was used for assessing exclusive breastfeeding of the infants.

The qualitative data were collected using text writing by a reporter and video recording to increase the consistency during synthesis and transcription. The author of this research guided the discussion using some introduction questions.

### 2.3. Operational Definition

Exclusive breastfeeding: exclusive breastfeeding means if the child takes only breast milk and no additional food, water, or other liquids (with the exception of medicine and vitamins, if needed) for the duration of 6 months [1, 22].

Complementary feeding: the provision of foods or liquids (other than breast milk) along with breast milk from the six month of life to the 24th month [23].

### 2.4. Data Processing and Analysis

The collected data were checked for completeness and coded manually and entered into Epi Info version 3.5.3. Statistical Package for Social Science Students (SPSS) version 23 was used for advanced analysis. Descriptive statistics of sociodemographic characteristics and the prevalence of exclusive breastfeeding were presented using tables. Binary logistic regression was carried out to identify factors associated with the exclusive breastfeeding practice. First, bivariable logistic regression was performed on each independent variable with the outcome variable. Then, those variables with a *P* value <0.2 were fitted in the final multivariable analysis to control confounders. The strength of the association was measured using odds ratio and 95% confidence intervals. Statistical significance was declared at a *P* value <0.05. Variables that were explored during FGD were synthesized thematically. But, only variables that were different from the variables explained by the quantitative studies were discussed.

## 3. Results

### 3.1. Sociodemographic Characteristics

A total of 408 mothers with a child less than two years of age participated in this study, giving a 96.4% response rate. Three hundred and sixty-four (52%) mothers were between 25–34 years of age. Approximately 89.2% of mothers were married, 62.7% were housewives, and 65% were Muslim religion followers. Of the participants, 15.4% and 43.8% were either unable to read and write or able to read and write but did not have formal education, respectively. Only 7.3% of mothers had less than 1000 Ethiopian birr monthly income at the household level (Table 1).

### 3.2. Participants of the FGD

Six focus group discussions were conducted among mothers and fathers who had children aged six months or above. The focus groups were held in three districts using a discussion guide. The group arrangement was designed by gender. Three FGDs were comprised of mothers, while the rest were fathers of the children. Twenty-nine women who were actively breastfeeding and 23 fathers who were from active breastfeeding families were invited to attend the focus group sessions. The group formation was mothers with a child aged six months or above (*g*_1_*n*_1_=9), female (*g*_2_*n*_2_=8), female (*g*_3_*n*_3_=12), fathers (*g*_4_*n*_4_=7), fathers (*g*_5_*n*_5_=8), and fathers (*g*_6_*n*_6_=8). There were no other criteria used for group formation other than gender.

The mean age of the fathers was 32 and females' was 28 years. The age ranged from 19 in female to 41 in male participants.

Twenty-four mothers and eight fathers reportedly heard about exclusive breastfeeding in different information sources. All the invitees (29 women and 23 men) survive by the traditional farming method. Only 7 females and 13 males had formal education.

The researcher facilitated each session, which lasted approximately 1 hour for each session.

### 3.3. Maternal Health Services Utilization and Related Characteristics

Of the 408 study participants, 342 (83.8%) mothers attended an ANC follow-up. Of those mothers who had ANC, 212 (61.9%) had four and above visits. Similarly, out of those who had an ANC follow-up, four-fifth 298 (87.1%) of the mothers received counseling about the importance and practice of exclusive breastfeeding (Table 2).

### 3.4. Breastfeeding Practice of Mothers with Children Aged Less Than Two Years

Two hundred and sixty-eight (65.8%) of 408 mothers put their newborns to breastfeed within one hour of birth. But, only 30 (7.3%) of the mothers have initiated breastfeeding after one day. Three hundred and fifty-one (86%) of 408 mothers exclusively breastfed their children for the first three days of birth. In this study, the prevalence of exclusive breastfeeding practice was 77.5% [95% CI: 73.5, 81.5%]. This finding was computed from mothers who had a child aged six months and above during data collection (Table 3).

### 3.5. Factors Associated with the Exclusive Breastfeeding Practice

After adjusting confounding variables in multivariable analysis, marital status, educational status, occupation, ANC follow-up, birthplace, and breastfeeding counseling of mothers were the independent predictors of the exclusive breastfeeding practice.

Married mothers were 2.6 times more likely to breastfeed their child exclusively, AOR = 2.57 [95% CI: 1.68–5.54], compared to unmarried mothers.

Likewise, mothers who had antenatal care follow-up had four times more odds of following the exclusive breastfeeding practice, AOR = 4.11 [95% CI: 2.66–11.17]. Mothers who delivered at health institutions four times more exclusively breastfed their child, AOR = 4.07 [95% CI: 2.99–10.72].

Regarding counseling, mothers who received breastfeeding counseling during their ANC follow-up had two times more odds of following exclusive breastfeeding compared to their counterparts, AOR = 1.96 [95% CI: 1.12–4.73].

On the other hand, employed mothers reduced exclusive breastfeeding by 78%, AOR = 0.22 [95% CI: 0.16, 0.56], compared to unemployed/housewife mothers. Concerning education, mothers who were unable to read and write were 89% less likely to practice exclusive breastfeeding than mothers with secondary and above educational status (Table 4).

### 3.6. Factors Explored by FGD

A lesser number of the FGD members (10 fathers) reported that they support their wives during child feeding practice. More mothers than fathers were happy with the discussion to help one another with child feeding practice.

Based on the FGD result, culture, outdoor jobs, and the major responsibility of mothers for child rearing and household jobs were the main factors being barriers for exclusive breastfeeding in rural communities of Ethiopia.

Although this discussion was performed in the Ethiopian community with specific local cultural beliefs, I believe that the principles and results found can be generalized to much of rural Ethiopia. This is because almost uniformity exists in communities whose economies are based on the production from the farm.

In Ethiopia, rural women have a belief that they should let their child taste every food that the mothers tasted. This is because their child will be harmed by Kolle (Kolle to mean invisible spirit that can harm if they did not do what this spirit needs) if they did not give food that they tasted for their children. They have a belief to touch the child's body, especially the lip, with the food if the child is asleep during their feeding. They give any homemade food to the child. They are not worried to give formulas. This belief decreases the exclusive breastfeeding of the children. “It might harm the babies from feeding them, but it might not harm more than the Kolle will harm them. It is our belief that comes from our grandparents and ancestors to make the babies taste the food that we, the parents, tasted (*g*_2_,  *p*_7_).”

The economic situation of women also is a barrier to exclusive breastfeeding. Many women stated that they could not maintain exclusive breastfeeding due to their work. Their daily work is outdoor farming, which is far from their house. Daily, they walk long distances to and from their fields. During the time away from home, the baby is left in the care of grandparents or other elder children. According to the age of the baby, cow's milk/butter, eggs, and fruits without formula are given when the baby cries to induce sleep until the mother returned from outdoor work. Some women expressed awareness of the importance of exclusive breastfeeding. “The baby may be benefited if exclusively breastfed. However, I cannot strictly exclusively breastfed because of our economic status. Therefore, I enforced to give homemade foods to a child without formula (*g*_3_,  *p*_2_).”

Almost all of the mothers who did not exclusively breastfeed their children agreed that indoor works and child rearing are the responsibilities of them. Household food preparation, house cleaning, cloth washing, rearing the children, and even washing the legs of the husband are the duties of the mothers. Recently, some of the duties are being shared by both the fathers and mothers. “If I have outdoor work, I will go and work for a long time. After return, I also have a responsibility to prepare food to feed the household members. During this time, I cannot exclusively breastfeed my child (*g*_1_,  *p*_8_).” This idea was shared by the fathers' discussion member. “I feel that we are making mistakes. Almost all of the indoor jobs are done by our wife. Similarly, they share our outdoor works, but the husbands did not share indoor jobs. These made them unable to breastfeed exclusively (*g*_6_,  *p*_2_).”

“*g*_5_,  *p*_5_: I am 38 years old. I and my wife have three children. In our life, I did not support my wife during breastfeeding. Instead of supporting her, I was shouting at her if one of our children cried. The health extension workers told us to support our wives. But, there is still husbands' superiority. This might be an obstacle for the exclusive breastfeeding practice.” Likewise, some men assumed being inferior if some else saw them during supporting their wife. “I tried to support my wife in indoor works, which will result in increasing exclusive breastfeeding. But, culturally, we believe that we are inferior if someone else sees us during indoor job sharing and child rearing (*g*_4_,  *p*_3_).”

The other factors mentioned by the FGD had similar themes and ideas with the quantitative findings of this research.

## 4. Discussion

Breast milk is an irreplaceable natural food for a newborn baby. It helps the babies to grow properly and protects them from infection.

This study revealed that the prevalence of exclusive breastfeeding was 77.5% [95% CI: 73.5–81.5%]. Despite its need, the prevalence of exclusive breastfeeding in this study was not satisfactory. This finding is comparable with the study in Addis Ababa (81%) [24], Ethiopian HSDP IV target level of 70% [25], and Debre Markos, Ethiopia, 60.8% [21]. However, the prevalence is higher than in the Ethiopian demographic health survey (EDHS) 16 data (58%) [10], Motta town [17], the global EBF estimate (35%), Nigeria (20%), Brazil (58%), and Bangladesh (34.5%) [1, 4, 11, 19]. This discrepancy might be due to sociodemographic variations, methodological differences, and sample size incomparability of the studies.

Concerning factors associated with exclusive breastfeeding practices, the mothers' level of education, marital status, occupation, ANC follow-up, place of delivery, and whether they received counseling about breastfeeding practice during the ANC visit were statistically significantly associated with the exclusive breastfeeding practice. Accordingly, mothers who were unable to read and write were less likely to practice exclusive breastfeeding compared to mothers of secondary education level. This result is discordant when compared to a study in Ethiopia [26]. The possible explanation for this difference might be due to the sample size difference.

After controlling the confounding variables, employed mothers were less likely to exclusively breastfeed than mothers who were housewives. This result is similar to studies in Awi Zone [27], Northwest Ethiopia [28], Debre Markos [21], Malaysia, [29, 30], Cameroon [31], and Ghana [32, 33]. This might be because women who spent their time at home are more frequently in contact with their child. On the other hand, employed mothers may not have frequent contact with their infants. This will hamper effective the exclusive breastfeeding practice. If they had support in the workplace to support their milk supply and produce milk for the infant when at work, they could continue to breastfeed. Therefore, the women would need to be given break time and a place to express their milk while at work.

This study revealed that mothers who attend an antenatal care follow-up were more likely to exclusively breastfeed. This is supported by a nested case-control study in Northwest Ethiopia [34] and Jimma [35] and the standard recommendation of breastfeeding guidelines [36, 37]. However, ANC has no association with the EBF practice according to a study in Motta town [17]. This difference might be attributed to the study year and the study population included.

Mothers who gave birth in a health institution were four times more likely to breastfeed their child when compared to those who gave birth at home. This finding is in agreement with the studies conducted in Bahir Dar [33] and Ghana [32]. This could be because mothers who give birth in the institution had more opportunities to be counseled about the benefit of breastfeeding by health-care providers. In contrast to this, a study conducted in Motta [17] indicated that the birthplace does not associate with the exclusive breastfeeding practice. This discrepancy might be due to the study period, study populations, and sociocultural differences.

Receiving breastfeeding counseling during antenatal care service was found to facilitate the exclusive breastfeeding practice. This is in parallel with studies conducted among low-income Latinos in the United States [38], Nigeria [39], and Debre Markos [21]. This implies that health education and counseling increase mothers' knowledge about the benefits of EBF.

Moreover, culture was one of the independent predictors of exclusive breastfeeding in this study. The study showed that mothers had a culture of giving different foods such as water, coffee, and fresh butter. This finding is in line with studies in Afar [40] and rural Ethiopia [41] and a study about exclusive breastfeeding measurements and indicators in Israel [42]. This similarity might be since most Ethiopian mothers believe that their child will be affected by the devil if a child does not taste a portion of food that the mothers tasted (visual witness from elders and FGD).

This study could have the following limitations. First, health institutions-related factors and special deference are not assessed. Second, since this study included mothers with children up to two years of age, recall bias might under- or overestimate the prevalence of exclusive breastfeeding. Third is the cross-sectional nature of the study design used, which could reveal poor causal establishment. Finally, the investigator recommends the researchers quantify the qualitative aspects explored by this study.

## 5. Conclusions

This study revealed a substantial prevalence of breastfeeding compared to the EDHS, Ethiopia report. Being married, having antenatal follow-up, institutional delivery, and counseling about breastfeeding during antenatal service were the variables that increase the exclusive breastfeeding practice. Unable to read and write and being employed were the negative predictors of exclusive breastfeeding. Therefore, strengthening ANC service, institutional delivery, and educating mothers and providing maternity leave will improve the breastfeeding practice. Moreover, the researcher noted that healthcare professionals, planners, and policy-makers might use the result of this study in guiding evidence-based decision-making regarding improving the exclusive breastfeeding practice. Finally, an employer-based program to support exclusive breastfeeding among working mothers should be endorsed and implemented.

## Figures and Tables

**Table 1:** Sociodemographic characteristics of mothers and children (*n* = 408) in Ethiopia, 2019.

Variable	Category	Frequency	Percentage (%)
*Age of the mother*	15–24	115	28
	25–34	212	52
	35–44	81	20

*Marital status of the mother*	Married	364	89.2
	Single	9	2.3
	Divorced	11	2.6
	Separated	15	3.8
	Widowed	9	1.9

*Religion of the mother*	Orthodox Christian	132	32.3
	Muslim	265	65
	Protestant^*∗*^	11	2.7

*Occupational status of the mother*	House wife	256	62.7
	Employee	150	36.9
	Merchant	2	0.4

*Occupational status of the husband*	Farmer	158	38.8
	Government employer	226	54.6
	Merchant	24	5.8

*Educational status of the mother*	Unable to read/write	63	15.4
	Able to read and write	179	43.8
	Primary education	96	23.5
	Secondary education	70	17.3

*Monthly income*	<1000	19	7.3
	1001–2500	117	45
	>2500	124	47.7

*Sex of the child*	Male	225	55.1
	Female	183	44.9

*Age of the child (month)*	<6	80	19.8
	>6	328	80.2

**Table 2:** Maternal and child health service utilization characteristics of study participants in Ethiopia, 2019.

Variable		Frequency	Percentage
*ANC visit*			
	Yes	342	83.8
	No	66	16.2

*ANC visits (N* *=* *342)*			
	One	10	2.7
	Two or three	120	35.2
	Four and above	212	61.9

*PNC follow-up*			
	Yes	312	76.5
	No	96	23.5

*Place of delivery*			
	Home	74	18.2
	Health facility	334	81.8

*Breastfeeding counsel during ANC*			
	Yes	298	87.1
	No	46	12.9

*Culture(ingesting butter at birth, making the child to test foods what the mother took, etc.) before six months age*			
	Yes	218	53.4
	No	190	46.6

**Table 3:** Breastfeeding practice of the mother having a child less than 2 years of age, Ethiopia, 2019.

Practice of the mother for child feeding	Category	Frequency	Percentage
*Breastfeeding initiation*			
	Immediately	268	65.8
	After 1 hour	110	26.9
	After a day	30	7.3

*Gave food (water, homemade nonformula foods, and cow's milk & butter)other than breast milk in the first 3 days of birth*			
	Yes	57	14
	No	351	86

*Types of food given*			
	Plain water	27	47.2
	Sugar solution	19	33.3
	Cow's milk	11	19.4

*No food given other than breast milk before the sixth month (N* *=* *328)?*			
	Yes	255	77.5
	No	73	22.5

*When do you usually feed the child?*			
	When the child likes to have	307	75.3
	When the child cries^*∗*^	101	24.7

*No. of breast milk feedings per day*			
	<8	147	36.2
	8–12	227	55.8
	>12	34	8.1

*Age of the child in months when breastfeeding was stopped*			
	<16	119	29.2
	17–23	185	45.3
	>23	104	25.5

*Start complimentary feeding practice at the sixth month (N* *=* *209)*			
	Yes	185	88.6
	No	24	11.4

^*∗*^Breast engorgement and when the mother was free of work are included.

**Table 4:** Factors associated with the exclusive breastfeeding practice among mothers with children aged less than two years in Ethiopia, 2019.

Variables		EBF practice			
		Yes	No	COR (95% CI)	AOR (95% CI)
*Marital status*	Married	238	116	3.01 (1.56, 5.80)	2.57 (1.68, 5.65)
	Not married	17	25	1	

*Educational status*	Unable to read/write	17	46	0.72 (0.34, 0.1.27)	0.11 (0.06, 0.99)
	Able to read and write	89	90	0.48 (0.27, 0.86)	
	Primary education	50	46	0.53 (0.28, 1.01)	
	Secondary education	47	23	1	1

*Occupation*	House wife	200	67	1	1
	Employee	55	86	0.21 (0.13, 0.33)	0.22 (0.16, 0.56)

*ANC follow-up*	Yes	238	99	6.92 (3.80, 12.61)	4.11 (2.66, 11.17)
	No	17	49	1	1

*Birth place*	Health institution^*∗*^	232	102	5.04 (2.92, 8.69)	4.07 (2.99, 10.72)
	Home	23	51	1	1

*PNC follow-up*	Yes	227	85	6.48 (3.91, 10.82)	
	No	28	68	1	

*Breastfeeding counsel during ANC*	Yes	230	63	2.77 (1.43, 5.35)	1.96 (1.12, 4.73)
	No	25	19	1	1

*Culture*	Yes	54	164	0.35 (0.23, 0.54)	0.23 (0.14, 0.46)
	No	91	99	1	1

## Data Availability

All the necessary data are included in the manuscript.

## Abbreviations

ANC: Antenatal care
AOR: Adjusted odds ratio
EBF: Exclusive breastfeeding
HSDP: Health sector development plan
OR: Odds ratio
PNC: Postnatal care.

## Supplementary Materials

Supplementary Materials Supplementary 1. English Version Questionnaire. STROBE checklist for cross-sectional Studies.
