# Supplementary material for: Exclusive Breastfeeding and Normative Belief among Rural Mothers in Ethiopia, 2019: A Cross-Sectional Survey Embedded with Qualitative Design
Source: Obstet Gynecol Int. 2021 Mar 16;2021:5587790. doi: 10.1155/2021/5587790 (PMC7987461; doi:10.1155/2021/5587790)
Supplement: Supplementary Materials — Supplementary 1. English Version Questionnaire. STROBE checklist for cross-sectional Studies. [file 5587790.f1.zip › 5587790.f1/Supplement 1.docx]

QUSTIONAURIE

**Part I: Socio-Demographic characteristics**

| 1.1 | Age in year at present ----------- |  |
| --- | --- | --- |
| 1.2 | Marital Status | 1. Married  2. Single  3. Divorced  4. Separated  5. Widowed |
| 1.3 | Religion | 1. Orthodox  2. Muslim  3. Protestant  4. Catholic  5. Other specify ……………. |
| 1.4 | Ethnicity | 1 Amhara  2. Oromia  3.Tigray |
| 1.5 | Respondent occupation | 1. Housewife 2. Merchant 3. Farmer 4. Daily labor 5. Student 6. Others (specify)….. |
| 1.6. | What is the main occupation of your husband | 1. Farmer 2. Daily labor 3. Merchant 4. Governmental employee 5. Others (specify)….. |
| 1.7 | Respondent educational status | 1. Unable to read and write 2. Can read and writes 3. Primary education (1-8) 4. Secondary education and above |
| 1. 8 | For those married husband educational status | 1. Unable to read and write 2. Read and writes   3. Primary education (1-8)  4.Secondary education and above |
| 1.9. | Monthly house hold income | ----------------------------(in Ethiopian Birr) |

**Part II. questions on maternity experience of the youngest child**

| 201 | How many children do you have now? | Number of children _____ |
| --- | --- | --- |
| 202 | Have you attended Antenatal clinic in any health facility while you were pregnant with the last Child? | 1. Yes_______ 2. No_______ |
| 203 | If question 2.1 answer is “yes” how many times? | Number of follow up __________ |
| 204 | Where did you give birth your last child? | 1. Home 2. Hospital 3. Health center |
| 205 | Following your last delivery have you attend postnatal clinic in any health facility? | 1. Yes 2. No |

**Part III. Questions about breast feeding**

| 301 | How long after birth did you first put the child to the breast? | 1. Immediately 2. Afterhours 3. After Day 4. Don’t know |
| --- | --- | --- |
| 302 | Within the first three days did you give any other than breast milk (colostrum?) | 1. Yes 2. No |
| 303 | Before six months, did you give other than breast milk for a child ? | 1. Yes 2. No |
| 304 | If question 303 answer is “Yes”, what did you then feed the child? | 1. Plain water 2. Sugar solution 3. Cow milk 4. Butter 5. Others |
| 305 | Are you still breast feeding the child? | 1. Yes 2. No |
| 306 | How many times did you breast-feed last day? | 1. Number________ 2. Do not know |
| 307 | When did you usually breast feed the youngest child? | 1. When the child likes to have 2. When the child cries 3. When breast engorge 4. Other (Specify)_____ |
| 308 | Starting from the date of birth up to 6 months, what did you feed your child? | 1. Nothing other than breast milk is fed 2. Water/tea c 3. Cow’s milk 4. Cereal based fluid 5. Adult food |
| 309 | When did you offer the child with additional diet beside your breast milk?(age of child in months). | Months____  Do not know____________ |

**Part IV. Questions on advice and support of breast feeding**

| 401 | When you were pregnant or in the period after delivery of the last child, have you ever been informed /advised about breast feeding by health care provider? | 1. Yes___ 2. No____ |
| --- | --- | --- |
| 402 | Whoever gave you help or advice how to start and continue breast feeding your Child? | 1. Husband 2. Friends/Neighbor 3. Health care providers 4. Other (specify)___ |
| 403 | In your opinion what should be the role of husband in breast feeding? | 1. Know the advantage of breast feeding and give advice 2. Give economic support 3. support and Involve on home activities 4. Don’t know |
| 404 | Do you feed an infant every food that mother tasted? | 1. Yes 2. No 3. I do not know |
| 405 | If question 404 answer is “Yes”, why? | 1. Love 2. Culture 3. Religious guide |
